# Detecting cardiac pathologies via machine learning on heart-rate variability time series and related markers

**DOI:** 10.1038/s41598-020-64083-4

**Published:** 2020-06-01

**Authors:** Elena Agliari, Adriano Barra, Orazio Antonio Barra, Alberto Fachechi, Lorenzo Franceschi Vento, Luciano Moretti

**Affiliations:** 1 Dipartimento di Matematica “Guido Castelnuovo”, Sapienza Università di Roma, P. le A. Moro, 00185 Roma, Italy; 2 Dipartimento di Matematica e Fisica “Ennio De Giorgi”, Università del Salento, Via per Arnesano, 73100 Lecce, Italy; 3 Istituto Nazionale di Fisica Nucleare (INFN), Campus Ecotekne, Via Monteroni, 73100 Lecce, Italy; 4 Department of Environmental Engineering, University of Calabria (UNICAL-DIAM), 87035 Arcavacata, Cosenza Italy; 5 Politecnico Internazionale “Scientia et Ars” (POLISA), Largo Intendenza, 89900 Vibo Valentia, Italy; 6 Department of Cardiology “C. & G. Mazzoni”, Hospital (APH), Via degli Iris, 63100 Ascoli-Piceno, Italy

**Keywords:** Health care, Scientific data, Diagnostic markers, Complex networks

## Abstract

In this paper we develop statistical algorithms to infer possible cardiac pathologies, based on data collected from 24 h Holter recording over a sample of 2829 labelled patients; labels highlight whether a patient is suffering from cardiac pathologies. In the first part of the work we analyze statistically the heart-beat series associated to each patient and we work them out to get a coarse-grained description of heart variability in terms of 49 markers well established in the reference community. These markers are then used as inputs for a multi-layer feed-forward neural network that we train in order to make it able to classify patients. However, before training the network, preliminary operations are in order to check the effective number of markers (via principal component analysis) and to achieve data augmentation (because of the broadness of the input data). With such groundwork, we finally train the network and show that it can classify with high accuracy (at most ~85% successful identifications) patients that are healthy from those displaying atrial fibrillation or congestive heart failure. In the second part of the work, we still start from raw data and we get a classification of pathologies in terms of their related networks: patients are associated to nodes and links are drawn according to a similarity measure between the related heart-beat series. We study the emergent properties of these networks looking for features (e.g., degree, clustering, clique proliferation) able to robustly discriminate between networks built over healthy patients or over patients suffering from cardiac pathologies. We find overall very good agreement among the two paved routes.

## Introduction

Artificial intelligence (AI) is gaining a growing role in healthcare: in the last years, several devices and advanced algorithms have been successfully employed to assist medical workers (see e.g.^1–5^). Among the most important goals of this partnership between humans and machines is the wide accessibility (even in low-income and remote areas) to medical assistance and the reduction of the time needed to reach a diagnosis. Of course, in order for AI-based devices to analyze large amounts of information and make (fast and correct) decisions, they first need to undergo a suitable training^6–9^. Basically, during training, a machine-learning model is exposed to examples and its internal parameters are tuned accordingly; once training is over, new data are presented to the model which then uses what it has learned to explain that data. For instance, a model meant to classify images of skin lesions as benign lesions or malignant skin cancer will be trained on a dataset of skin pictures from different patients, previously labeled as benign or malignant, through which the model learns to detect in the input image specific patterns that are hallmarks of malignancies. Clearly, the more accurate the training and the better the performance. Nowadays, an accurate training is in principle possible given that each patient generates large volumes of health data such as X-ray results, vaccinations, blood samples, vital signs, DNA sequences, current medications, other past medical history, and much more.

As mentioned above, a particularly important application of machine learning in a healthcare context is (digital) diagnosis (see e.g.^10–14^). Machine learning models can detect patterns (precursor) of certain diseases within patient electronic healthcare records and inform clinicians of any anomalies. Among the most successful examples so far, we mention its use in breast and skin cancer screening, in macular degeneration and diabetic retinopathy detection and in distinguishing bacterial and viral pneumonia on chest X-rays (see e.g.^15–17^). Notably, in this context training is often impaired by the relative sparsity of pathological examples for which the statistics is, luckily, typically lower than statistics over healthy examples.

In this work we focus on data concerning heart activity and we aim to apply machine learning tools to detect possible heart-related pathologies such as atrial fibrillation or congestive heart failure. The automatic prediction of pathological events from heart activity data has been intensively investigated, especially in the last decade (see e.g.^18–24^): its relative low-cost and non-invasive nature make it particularly promising.

Our dataset is made of Holter recordings on labelled patients (labels distinguish the kind of pathology affecting the patient, if any). The underlying idea is that the heart-rate variability (HRV), namely the variability in the time interval between heartbeats (which, to some extent, is perfectly physiological), may reveal patterns that are typical of heart-related pathologies. It is worth recalling that the HRV is usually measured in terms of the variation in the beat-to-beat interval and the common measure obtained from an Holter recording is the so-called RR variability (where R is a point corresponding to the peak of the Holter wave and RR is the interval between successive Rs, that in humans is ~10^3^ ms). A preliminary statistical investigation on these raw data allows us to see that RR intervals display heavy-tailed distributions. A more convenient, corse-grained description can then be achieved in terms of a set of markers widely used in the cardiology research community^25^. These are also suitable candidate as inputs for a machine learning network. The examples available in our database are therefore used for the training and the validation of a multilayer feed-forward network which turns out to be able to classify patients into four different categories: healthy patients (i.e., control group), patients suffering from atrial fibrillation, or from congestive heart failure, or from other disease.

Beyond these AI-based methods for distinguishing between healthy and pathology heart-beats, there exist – and have proved to be successful – other methods based on statistical mechanics. In particular, we mention approaches focusing on correlations displayed by beat-to-beat fluctuations, such as the detrended fluctuation analysis^26–29^, and on multifractality in heartbeat interval time series^30,31^. Within this wider scenario, in the second part of this paper, we look for consistency following a totally different investigation route based on network theory^32–35^. More precisely, we assign to each RR-series a node in a network and we tie nodes together according to a similarity measure between series. Then, we highlight that networks built on healthy patients (i.e., control group), on patients suffering from atrial fibrillation, or from congestive heart failure exhibit different topological features. Overall, the results of these two routes are in very good agreement and the potentialities shown by these kinds of approach should motivate the establishment of suitable repositories and clouds aimed for an accurate training of these networks and algorithms.

## Results

### Data description: from time series to markers

This research has been accomplished as part of the project *MATCH* (Mathematical Advanced Tools to Catch Heart-rate-variability), a scientific cooperation among Ascoli-Piceno Hospital (APH), International Polytechnic “Scientia et Ars” (POLISA), University of Salento, and University of Calabria. The initial database used in the present analysis consists in nominal 24 h Holter recordings of $M=2829$ patients hospitalized in APH, whose data were managed at POLISA, in the period 2016–2019.

Patients are divided into 6 main classes: healthy (H), suffering from atrial fibrillation (AF), from congestive heart failure (i.e. cardiac decompensation, CD), from diabetes (DIAB), from hypo- or hyperthyroidism (TIR), or from hypertension (TENS). Following Holter recordings, each patient is associated to an RR time-series, namely a series of temporal intervals between two consecutive heart-beats. For instance, being ${t}_{n}$ the time for the $N$-th beat and $L$ the total number of RR intervals in a given time-series, the ${n}^{th}$ RR-value reads as

2.1 $${{\rm{RR}}}_{n}={t}_{n+1}-{t}_{n},\,{\rm{for}}\,n=1,\ldots ,L-1.$$

**Figure 1:**
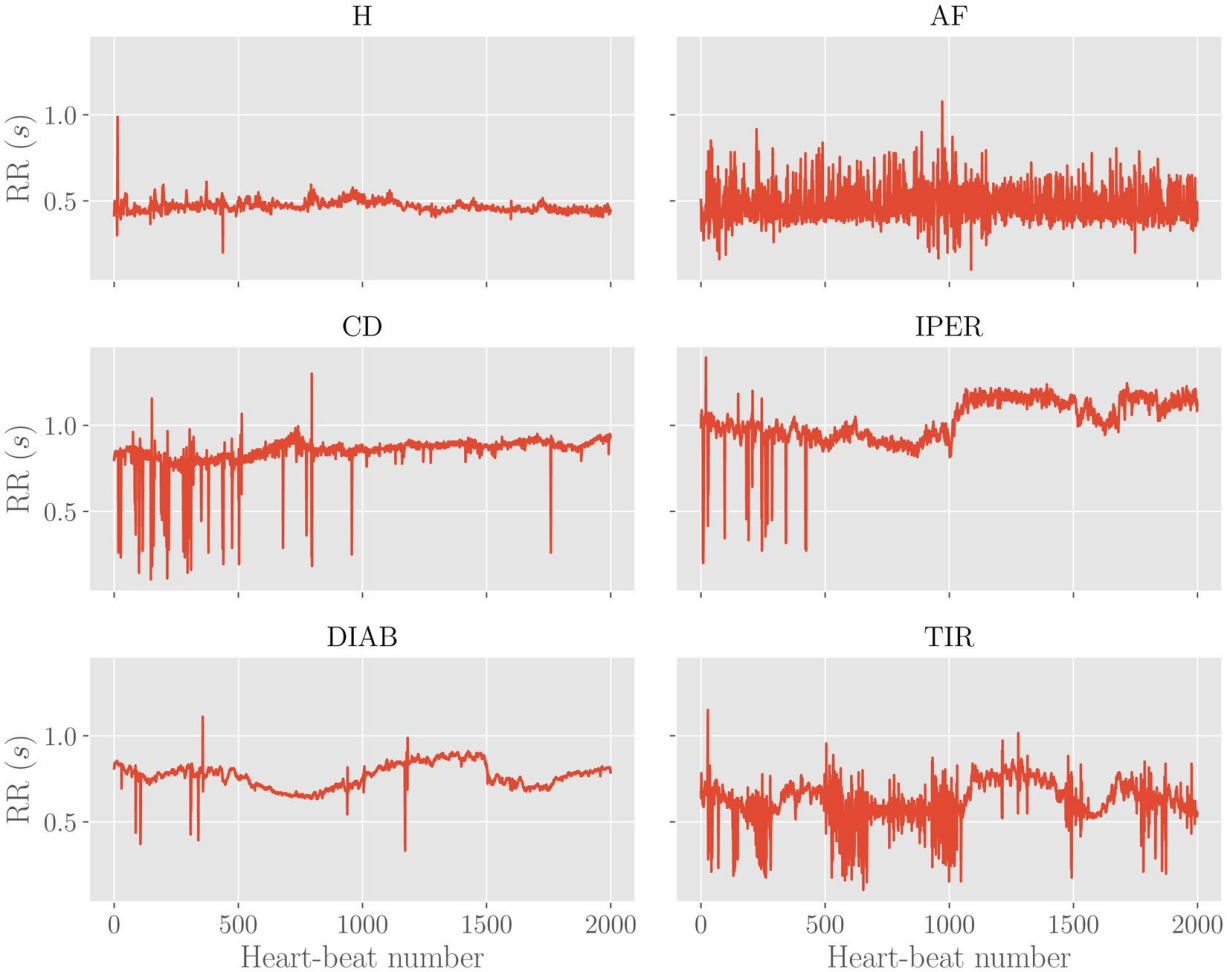
Examples of RR time-series for different classes. Each plot shows the first 2000 points (i.e. heart beats) with the corresponding RR intervals (in seconds) for the various classes considered.

Examples of these series for the different classes are reported in Fig. 1.

Another possible measure for heart-rate variability is given by the Beats-Per-Minute (BPM) sequence, which can be obtained by counting how many RR intervals occur in a single minute.

**Figure 2:**
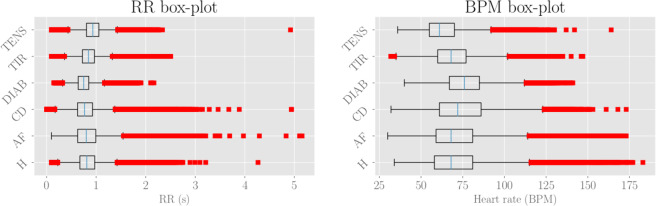
Left: RR box-plot (left panel) and BPM box-plot (right panel) for each subclass. The boxes represent the interquartile range, while the blue line is the median. Horizontal lines are the fliers extending from the boxes ends up in the whiskers, and finally red dots represent outlier points. The sample sizes are, respectively, 600 (H), 560 (AF), 232 (CD), 217 (TIR), 161 (DIAB), 113 (TEN).

As a preliminary statistical insight, we look at the distributions of RR’s and BPM’s values. By merging data pertaining to patients belonging to the same class, we obtain as many box-plots as shown in Fig. 2. For each class, the median value (over all related patients) is denoted with the blue vertical line. The boxes extend from the lower to the upper quartile values, while the outer bars (whiskers) extend from the lowest to the highest non-outlier data (we recall that outlier points are observation falling outside the interval $[{Q}_{3}-\frac{3}{2}({Q}_{3}-{Q}_{1}),{Q}_{3}+\frac{3}{2}({Q}_{3}-{Q}_{1})]$, where ${Q}_{1}$ and ${Q}_{3}$ are, respectively, the lower and upper quartiles). For both RR and BPM data the structure of box-plots look quite similar in all subclasses, with outliers especially falling on the right side and suggesting that the underlying distributions exhibit a right symmetry with a heavy tail.

Beyond this description based on raw data, we can obtain a coarser one based on a set of 49 markers indicated by the European Society of Cardiology and the North American Society of Pacing and Electrophysiology (see e.g.^25^) to summarise the HRV within the RR and BPM series collected during an Holter recording (see also^18^). These markers are of different nature as briefly summarized hereafter (the exhaustive list is reported in Appendix A):

- **Linear markers pertaining to the temporal domain**, such as the mean value and the standard deviation of RR and of BPM data, the number of successive RR intervals that exceed a certain threshold, etc;
- **Linear markers pertaining to the frequency domain**, such as the frequency peaks, the absolute/relative/normalized powers in, respectively, the very low frequencies (VLF, i.e., $f\le 0.04\,{\rm{Hz}}$), the low frequencies (LF, i.e., $f\in [0.04\,{\rm{Hz}},0.15\,{\rm{Hz}}]$), and the high frequencies (HF, i.e., $f\ge 0.15\,{\rm{Hz}}$) ranges, etc.
- **Non-linear markers**, such as the standard deviations of the Poincaré plot, the approximate and sample entropies, the correlation dimension, etc.

As mentioned before, moving from a description in terms of the RR or BPM sequences to a description in terms of markers implies a coarsening and, as a consequence, the clinical picture of the $n$-th patient, with $n=\mathrm{1,}\ldots ,M$ is now simply represented by a vector ${{\boldsymbol{x}}}_{n}=({x}_{n}^{(1)},\ldots ,{x}_{n}^{(49)})\in {{\mathbb{R}}}^{49}$, where the $i$-th marker ${x}_{n}^{(i)}$, with $i=1,\ldots ,49$, is a scalar quantity.

For any $(i,n)$, we can compute the marker value ${x}_{n}^{(i)}$ from the raw RR time-series by means of Matlab-based software^36^. Then, the average and the standard deviation (over the whole population making up the database) follow, respectively, as

2.2 $${\bar{x}}^{(i)}=\frac{1}{M}\,\mathop{\sum }\limits_{n=1}^{M}\,{x}_{n}^{(i)},$$

23 $${\sigma }^{(i)}=\sqrt{\frac{1}{M}\,\mathop{\sum }\limits_{n=1}^{M}\,{({x}_{n}^{(i)}-{\bar{x}}^{(i)})}^{2}}.$$

Their definitions for $i=1,\ldots ,49$ are reported in Table 3 of Appendix A.

Despite the coarsening applied, the space ${{\mathbb{R}}}^{49}$ still exhibits a relative high dimension, which makes inference rather challenging. However, one can see that the 49 markers considered are not uncorrelated with each other. In fact, some markers present trivial relations as, for instance, the average of RR intervals and the average of the BPM. Therefore, it is convenient to preliminary study the correlations between markers in order to drop out redundant ones, yet preserving the whole information acquired. From a machine learning point of view, this analysis has the benefit of sensibly decreasing the number of free parameters to be tuned in the training procedure, so that over-fitting risks are effectively reduced. The correlation analysis will be performed in the next Section.

### Correlation analysis and dimensionality reduction

**Figure 3:**
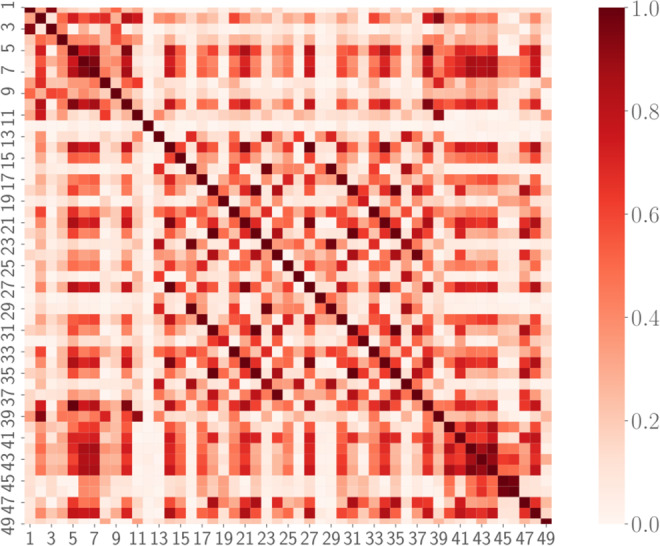
Correlation plot for the 49 standardized markers. The colormap refers to the absolute value of the Pearson correlation coefficient *C*_*ij*_, since we are only interested in the magnitude of possible correlations.

**Figure 4:**
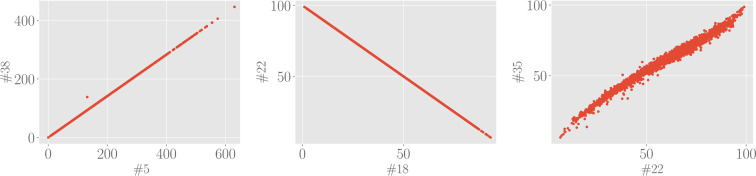
Examples of scatter plot for highly correlated markers. Left panel: Standard deviation of the Poincaré plot in the direction orthogonal to the identity line (#38) versus square root of the mean squared differences between successive RR intervals (#5) displays a perfect correlation with unitary Pearson correlation. Central panel: Normalized power of the LF band evaluated with FFT-based methods (#22) versus normalized power of the HF band evaluated with FFT-based methods (#18) displays a perfect anti-correlation with Pearson coefficient equal to −1. Right panel: Normalized power of the HF band evaluated with autoregressive methods (#35) versus normalized power of the LF band evaluated with FFT-based methods (#22) display a large correlation with Pearson coefficient equal to 0.992.

The simplest quantifier for correlation between marker $i$ and marker $j$ (with $i,j=1,\ldots ,49$) is the Pearson correlation coefficient ${C}_{ij}$ that reads as

24 $${C}_{ij}=\frac{{\rm{Cov}}({x}^{(i)},{x}^{(j)})}{\sqrt{{\rm{Var}}({x}^{(i)})\,{\rm{Var}}({x}^{(j)})}},$$

where

25 $${\rm{Cov}}({x}^{(i)},{x}^{(j)})=\frac{1}{M}\,\mathop{\sum }\limits_{n=1}^{M}\,({x}_{n}^{(i)}-{\bar{x}}^{(i)})({x}_{n}^{(j)}-{\bar{x}}^{(j)}),\,{\rm{Var}}({x}^{(i)})={\sigma }^{{(i)}^{2}},$$

are, respectively, the sample covariance and variance. Since we want to unveil (linearly) dependent markers, we will not care of the sign of the correlation but we will just look at the absolute value of the Pearson correlation coefficient, which is graphically represented in Fig. 3 for all the 49 × 49 possible pairs. By inspecting this plot, we see that there exists a non-empty set of mutually correlated variables: we report in Table 1 marker’s couples $(i,j)$ whose Pearson correlation coefficient is in magnitude higher than $|{C}_{ij}|\ge 0.990$. Examples of scatter plots for these highly-correlated markers are reported in Fig. 4. As remarked above, many of these correlations are somewhat trivial, for instance, this is the case for quantities in the frequency domain which are computed with FFT-based and with autoregressive methods. Since there is a negligible information loss if we discard one of two highly correlated markers, as a result of the analysis performed in this section, we can reduce the dimensionality of the marker space. In particular, we choose to discard eight markers: #22 (normalized power of the LF band evaluated with FFT-base methods), #27 (relative power of the VLF band evaluated with autoregressive methods), #29 (absolute power of the LF band evaluated with autoregressive methods), #31 (normalized power of the LF band evaluated with autoregressive methods), #35 (normalized power of the HF band evaluated with autoregressive methods), #38 (standard deviation of the Poincaré plot in the direction orthogonal to the identity line).

**Table 1:** Pearson correlation coefficients for highly correlated couples of markers.

*i*	*j*	*C*(*i*, *j*)
5	38	1
14	27	0.998
16	29	1
18	22	−1
18	31	0.992
18	35	−0.992
20	33	0.999
21	34	0.998
22	31	−0.992
22	35	0.992
31	35	−1

### Classification tasks by feed-forward neural networks

One of the goals of the current work is to evaluate whether observations based on Holter recording allow an automatic inference about the presence of pathologies like atrial fibrillation, congestive heart failure, diabetes, hypo- or hyperthyroidism, hypertension, etc. This task can be approached by several perspectives, from classical statistical inference methods to machine learning techniques. In any case, having large samples is a necessary condition for meaningful outcomes. As mentioned in Sec. 2.1, our databases is split into 6 main classes (H, AF, CD, DIA, T, TENS), of which only three (i.e., H, AF, CD) display a relatively large size. We therefore look for a tradeoff between statistical soundness and refinment in the emerging classification: in the following analysis, in order to avoid sparse classes, we slightly rearrange the initial database in order to generate two data-sets optimized for training classification of AF and CD patients solely, corresponding to (see Table 2)

- clinical data (markers) for healthy patients (H), patients suffering from atrial fibrillation (AF), patients suffering from other (not specified) diseases (O);
- clinical data (markers) for healthy patients (H), patients suffering from congestive heart failure (CD), patients suffering from other (not specified) diseases (O).

**Table 2:** Rearranged database composition.

Class	H/AF/O database	H/CD/O database
Healthy	600 patients	600 patients
Target disease	560 patients	232 patients
Other diseases	1669 patients	1997 patients

Note that the healthy patients in the two databases coincide, while the O class for the first database partially overlaps with the CD class of the second one (and, likewise, the class O for the second database may contain AF patient too). Since we analyze the two databases independently, this is not a problem, rather, this will allows us to double-check after classification (e.g., a patient targeted as AF in the first database should belong to the class O in the second database and a patient classified as CD in the second database should belong to the class O in the first database; breaking this rule would result in a fault-classification by the neural network).

Before proceeding, we perform a couple of tests to see if the rearranged dataset allows for some kind of data clusterization which may encourage further investigations via machine learning tools.

**Figure 5:**
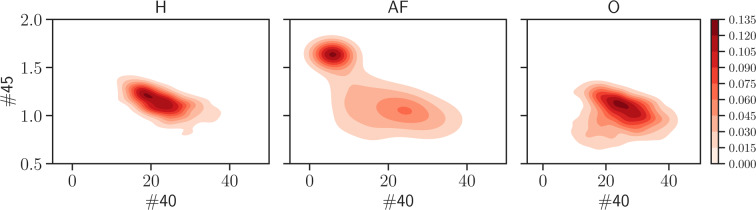
These panels display the joint probability distribution ${P}^{{\rm{class}}}({x}^{(i)},{x}^{(j)})$ for the couple (40, 45) of markers, obtained from the H (left), the AF (middle) and the O (right) classes. As highlighted by the shared colormap on the right, darker regions are those more more likely. Remarkably, the AF population tends to condense in a region quite far from the one characteristic of H and O patients, suggesting that they can be effectively distinguished from the ${\rm{H}}+{\rm{O}}$ bulk.

First, we look at the joint distribution ${P}^{{\rm{class}}}({x}^{(i)},{x}^{(j)})$, where “class” can be H, AF, O (or H, CD, O), which is obtained by counting the number of patients belonging to the class considered and displaying value ${x}^{(i)}$ for marker $i$ *and* value ${x}^{(j)}$ for marker $j$. In particular, we look at the joint probability by distinguishing between the classes H/AF/O and check whether some clusterization occurs in some 2-dimensional plane in the marker’s space (which can be a useful prerequisite in order to obtain a meaningful classification of the patients). Indeed, this turns out to be the case, as shown in Fig. 5, where one can see that the projections onto given planes in the high dimensional data space of the joint distribution evaluated for the AF class has a clearly different clusterization with respect to the H and O classes.

**Figure 6:**
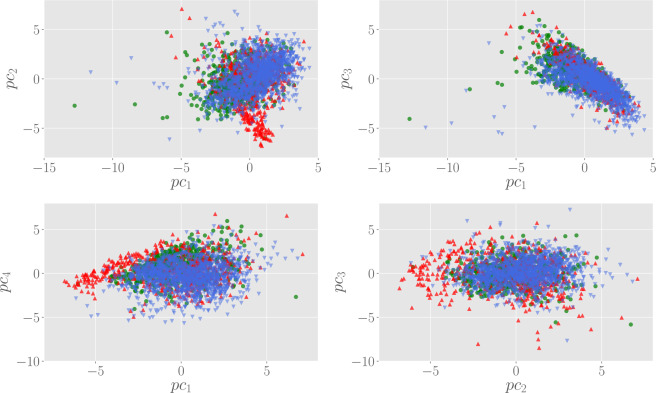
Scatter plots in the space spanned by the first principal components. Here, green circles represent H patients, while AF and O are, respectively, red up and blue down triangles. In general, AF patients tend to clusterize in a zone outside the cloud of the ${\rm{H}}+{\rm{O}}$ population.

A similar clusterization of AF patients can also be visualized in the space of the principal components. In particular, in Fig. 6 we show the scatter plot in the space spanned by the first four principal components, which overall encode for the 75% of the variability contained in the (standardized) marker data. From these plots, we can see that the population of H and O patients forms a wide cloud centered on the origin of the plane and distributed over a wide region, while AF patients tend to concentrate far from the origin, a feature that is clear in particular looking at the $p{c}_{1}$ vs $p{c}_{4}$ and $p{c}_{2}$ vs $p{c}_{3}$ scatter plots.

#### Enlarging the size of the markers via Principal Component Analysis soiling

Data augmentation is a popular and consolidated technique that allows improving classification and generalization in (deep) neural networks, whose training requires massive databases not always of immediate availability (as in the present case dealing with RR series of patients affected by particular diseases), see e.g.^37^. Indeed, its purpose is to synthesize new examples following the original data distribution; note that since such enriched database for training the machine can improve the generalization capabilities of the latter, it can be seen as an implicit and effective regularization. Data augmentation has already shown remarkable results in cross-cutting fields such as image processing^38,39^ or speech recognition^40,41^.

In general, in machine learning applications, we divide the database in two parts. The first one, referred to as the *training set*, contains a larger number of examples and it is used to infer the network parameters, while the second one, referred to as the *validation set*, is used to estimate the prediction performances of the model. Despite the encouraging results obtained from a statistical perspective in the previous sections, our database is rather small for a sound machine learning approach to the classification problem: techniques of data augmentation are needed in this case. The one adopted here is based on the work^42^, and consists in the augmentation of the data by introducing some noise in the principal components. More precisely, the augmentation algorithm is the following:

1. For each point in the dataset, multiply the first principal component $p{c}_{1}$ by a factor $\alpha$ drawn from a uniform distribution with support $(1-\epsilon /2,1+\epsilon /2)$, with $0 < \epsilon  < 1$. For each data point, this operation can be performed several times;
2. Propagate the new points back in the original data space (according to the associated rotation matrix).

**Figure 7:**
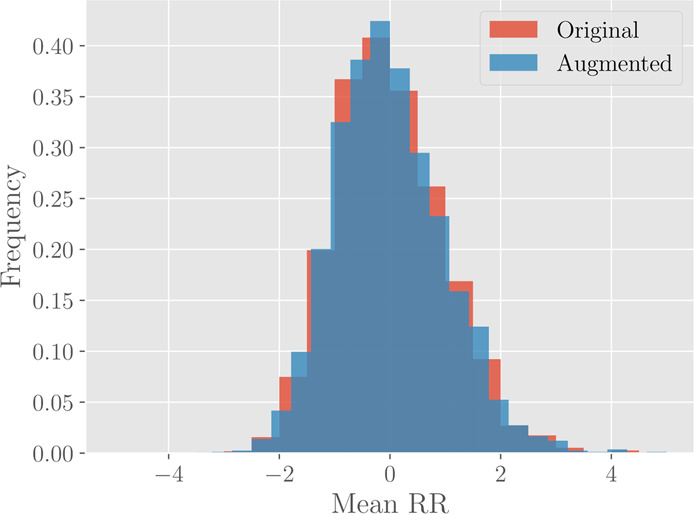
Histograms for the marker #1 (Mean RR) before (red) and after (blue) the data augmentation. The original histogram takes into account 2300 different patients, while the augmented one is drawn from 46000 different data points.

This procedure introduces some noise, but still preserves the variability structure of data. This fact can be visualized for instance by comparing the histograms of each marker before and after the data augmentation, as reported in Fig. 7. The augmentation is performed by choosing the perturbation amplitude $\epsilon =0.1$ and generating 20 new data points for each example in the original set. As a result, the validation set is augmented from 2300 initial patients to 46000 data points. As one can see from Fig. 7, the histograms of the marker before and after augmentation are nicely overlapped.

#### Neural network design and analysis

Given the results of the previous sections, we are now able to design neural network models for the classification problem. Of particular inspiration to this aim are the joint probability plots in Fig. 5, from which we find that there is a certain degree of separation between individuals with atrial fibrillation with respect to the remaining background (i.e., healthy people or patients with some other pathology). Therefore, we start developing an AF/NAF (Atrial Fibrillation vs Not Atrial Fibrillation) classifier for separating these two possibilities.

**Figure 8:**
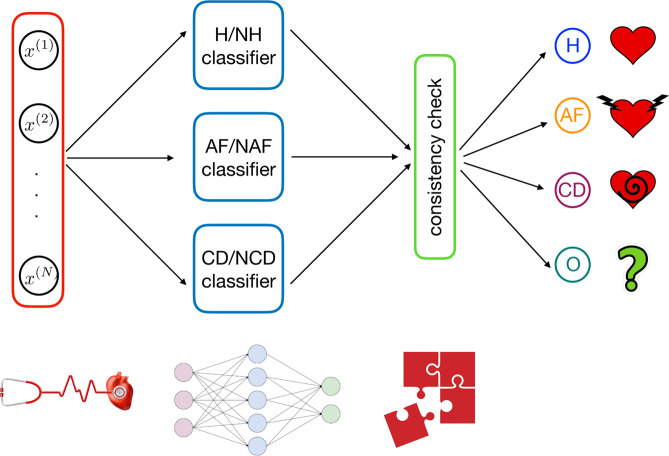
Overall architecture of the classifier developed in this work. From left to right: first, for a given example $n$, we evaluate the entries of the vector ***x***_*n*_ and we use this as input for the machine. The number of entries in the input vector is $N=41$. This input is then simultaneously passed to the H/NH block – which evaluates whether the example corresponds to a healthy unit or not – to the AF/NAF block – which evaluates whether the example corresponds to a unit displaying atrial fibrillation or not – and to the CD/NCD block – which evaluates whether the example corresponds to a unit displaying congestive heart failure or not. The outcomes stemming from this layer are compared checking for consistency; if this test is passed the outer layer provides the classification.

Then, analogous classifiers for H/NH (Healthy vs Not Healthy) and CD/NCD (Cardiac Decompensation vs Not Cardiac Decompensation) are also designed in such a way that the whole classifier is composed by these three building blocks, as reported in Fig. 8.

The main advantage of such an architecture is that each single classifier can be trained in separate and parallel way with respect to the others (moreover, this also allows to realized classifiers for specific tasks whether the performances are low). This modular scheme considerably reduces the computation time needed to find an optimal tuning of the parameters with respect to a monolitic architecture.

#### Training, generalization and classification performances

In this subsection we describe the functioning of each of the three blocks making up our model.

**Figure 9:**
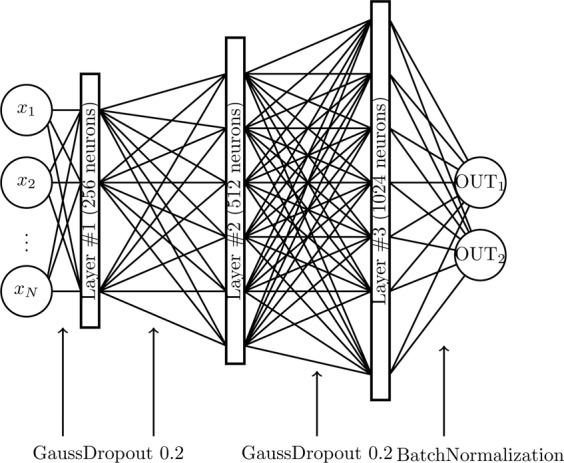
Neural network architecture of the single classifier. The hidden layers are composed by neurons with exponential linear units (ELU), while the output layer is composed by two softmax neurons. Before each hidden layer, a Gaussian Dropout operation is performed, while before the output layer a Batch Normalization is executed. The OUT_1,2_ neurons are chosen according to the specific task (i.e. H/NH, AF/NAF or CD/NCD).

**Figure 10:**
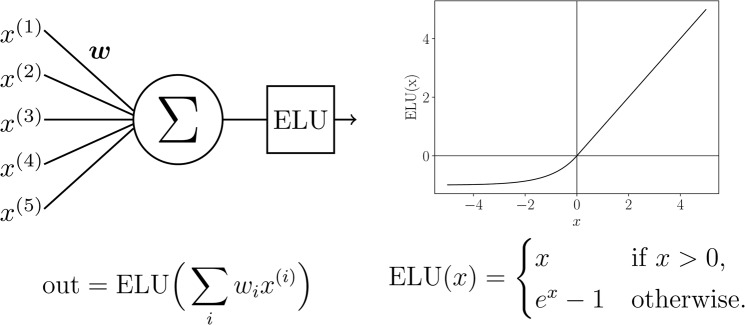
Schematical representation of ELU hidden units. In the left column, we give a schematical representation of neurons in the hidden layers. The inputs are summed according to the weights $w$, then the result is given as argument to the activation function (which is mathematically defined and depicted in the right column).

The neural model is realized using the Keras framework in Python; the hardware hosting the neural network is a double cluster composed by 16 CPU (all the cores work at a clock frequency of 3.0 Ghz) handing 216 GPU and equipped with a 32 GB RAM per cluster. Inputs (the markers describing the status of a given patient) are sent to the neural network which is composed by three hidden layers (made up of respectively 256, 512, 1024 exponential linear units, see Figs. 9 and 10). At each layer input, a Gaussian dropout (with value 0.2) operation is performed in order to avoid overfitting^43^. The signal outgoing from the third hidden layer is finally subjected to a Batch Normalization^44^, and then sent to the two output soft-max^45^ neurons (see caption of Fig. 9 for more details). We found experimentally that such a choice for network architecture and neuron’s activation function is a good compromise between generalization performances and training time.

During the training stage weights among neurons are tuned in order to minimize the categorical cross-entropy loss function ${\mathcal L}$ defined as

26 $$ {\mathcal L} =-\,\sum _{i}\,{t}_{i}\,\mathrm{ln}\,[f({s}_{i})],\,{\rm{with}}\,f{(s)}_{i}=\frac{{e}^{{s}_{j}}}{{\sum }_{j}\,{e}^{{s}_{j}}},$$

where ${s}_{i}$ is the stimulus acting on the $i$-th soft-max neuron and $f({s}_{i})$ is the related outcome to be compared with the true value ${t}_{i}$; minimization of ${\mathcal L}$ is achieved via a stochastic gradient descent method (both with momentum^46^ and Nesterov^47,48^ acceleration methods). As we work with labeled databases, the training stage is fully supervised, and it is split in two stages as explained hereafter. In the first stage, we present to the network a large and noisy version of the database (i.e., the one produced with the PCA-based augmentation criterion). This is the *pre-training stage*, in which the network gets *prepared* typically resting in a configuration close to the optimal one (i.e., the one related to the global minimum in the loss function landscape). Each pre-training stage is composed by 50 epochs, each epoch handling a mini-batch of 2000 example on which the gradient is computed and averaged. After that, the network is trained with the real database for 700 epochs with mini-batches of 300 examples for each of them.

After each epoch we evaluate the network performance in terms of the categorical accuracy, that is measured as the fraction of examples correctly classified by the network; the adjective “categorical” refers to the binarization of the network output as soft-max neurons actually provide an estimate for the probability of each classe (e.g., H versus NH, see Fig. 9) and the class finally selected is just the most probable. Notice that, after each epoch, accuracy is measured over the training set as well as the validation set.

**Figure 11:**
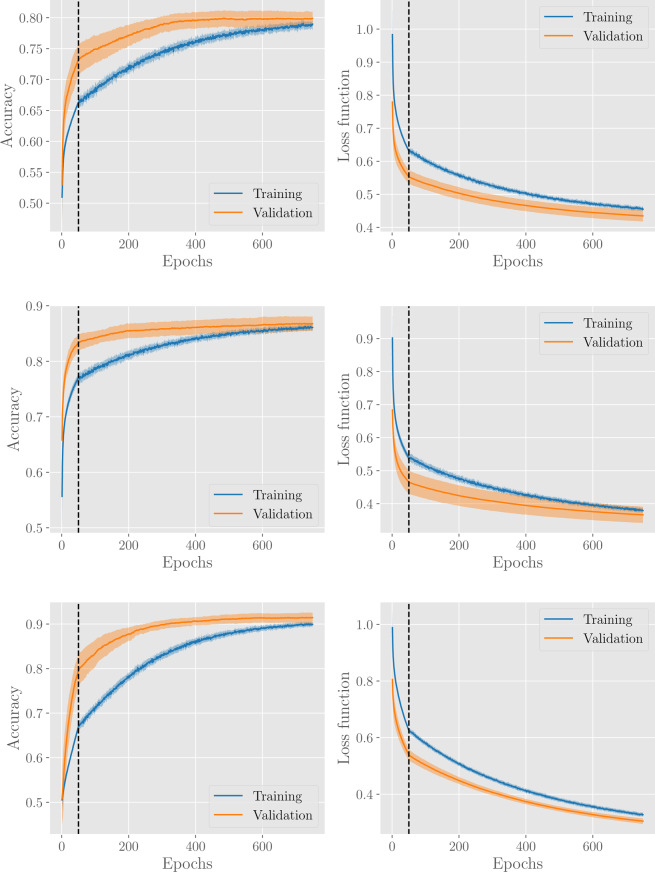
Results for the H/NH classifier (first row), AF/NAF classifier (second row) and CD/NCD classifier (third row). Panels in the left and in the right column show, respectively, the evolution of the accuracy and of the loss functions with the number of epochs. The vertical dashed lines denote the pre-training stages. Results are averaged over 20 different network training procedures, where, in each procedure, the dabase is split into a training set (containing 80% of examples, from the H, AF, CD, or O subsets respectively) and a validation set (containing the remaining 20% of examples). The solid line corresponds to the average over these procedures, while the coloured area around the curve highlights the standard deviation interval.

The evolution of accuracy and loss over epochs, for the H/NH, AF/NAF, and CD/NCD classifiers is shown in Fig. 11. In general, our learning procedure gives good performances with accuracy around 0.8–0.9 for all classification tasks. In particular, the training and the validation accuracies have a monotonic trend within the considered learning time and the former is always below the latter (this is a known effect due to dropout regularization, since during training the network deals with an incomplete representation of the data).

### Classification tasks via algorithmic network theory

Another route to heart failure’s classification could be paved by dealing directly with RR series, exploiting algorithms from network’s theory: these are particularly suitable^33–35,49^ as they allow introducing novel classes of network-based markers (e.g., degree centrality, maximal degree, clustering coefficient, betweenness centrality, reciprocity and cliques, vide infra).

The underlying idea is to consider a sample of $N$ patients within each of the highlighted categories (i.e. H, AF, CD) and associate to each of them a node of a graph ${{\mathscr{G}}}_{{\rm{H}},{\rm{AF}},{\rm{CD}}}$, then, for all the possible couples of nodes within this graph, we measure the similarity between the related time series. The similarity between the RR series ${{\bf{r}}}_{i}$ and ${{\bf{r}}}_{j}$ corresponding to nodes labelled as $i$ and $j$, respectively, provides the weight associated to the link connecting $i$ and $j$. Of course, for any choice of the sample of patients we obtain a different realization of the graphs ${{\mathscr{G}}}_{{\rm{H}},{\rm{AF}},{\rm{CD}}}$, and we are interested in any characteristic feature able to discriminate among the three classes and that is robust with respect to the sampling.

#### Graph realization via dynamic time warping

Similarity between patient RR-series can be defined in various way, but the key point is that two RR series are similar if they have comparable structure. Probably, the easiest choice would be in terms of the Euclidean distance between points in the two time series that occur at the same time

27 $$d({{\bf{r}}}_{i},{{\bf{r}}}_{j})=\sqrt{\mathop{\sum }\limits_{n\mathrm{=1}}^{\min ({L}_{i},{L}_{j})}\,{[{r}_{i}(n)-{r}_{j}(n)]}^{2}},$$

where we accounted for series of possible different length (${L}_{i}\ne {L}_{j}$). This is a good metric for similarity if both time series are in sync and move at exactly the same speed and time (i.e., all similar events in both time series occur at exactly the same time). However, when the series are out of sync this turns out to be a bad choice. In fact, in this case similar points in the two series could be stretched farther apart by time and the Euclidean distance would then get larger, suggesting, wrongly, that the series are becoming less similar.

To overcome this issue, we will adopt the so called “dynamic time warping” (DTW)^50,51^. This is an algorithm used to measure similarity between two sequences which may vary in time or speed. It works as follows:

1. Divide the two series into equal points ${n}_{1},{n}_{2},\ldots ,{n}_{\ell }$.
2. Calculate the Euclidean distance between the first point in the first series ${r}_{1}({n}_{1})$ and every point in the second series ${r}_{2}({n}_{i})$, $i=1,\ldots ,\ell$. Store the minimum Euclidean distance calculated.
3. Move to the second point ${n}_{2}$ and repeat stage 2. Move step by step along points and repeat stage 2 till all points are exhausted.
4. Repeat 2 and 3 but with the second series as a reference point.
5. Add up all the minimum distances that were stored and this is a true measure of similarity between the two series.

The time complexity of DTW algorithm is ${\mathscr{O}}({L}_{1}\times {L}_{2})$, where ${L}_{1}$ and ${L}_{2}$ are the lengths of the two input sequences. Assuming that ${L}_{1}\ge {L}_{2}$, the time complexity can be said to be ${\mathscr{O}}({L}_{1}^{2})$. Softwares designed to evaluate this distance often implement some optimizations in the algorithms in order to contain the computation time (see e.g.^52^).

As anticipated, according to the DWT similarity measure we derive a weight ${w}_{ij}$ between any pair of nodes. This is used to generate a fully-connected, symmetric weighted graph, where the weight associated to the link between the nodes corresponding to $i$ and $j$ is simply ${w}_{ij}$.

En route for the adjacency matrix, we proceed our analysis by applying an operation $f:{{\mathbb{R}}}^{+}\to \{0,1\}$, which makes the network un-weighted. A possible choice, determined by a parameter $k < N$, is given by

28 $${f}_{k}({w}_{ij})=\{\begin{array}{ll}1, & {\rm{if}}\,j\in {{\mathscr{N}}}_{k}(i)\\ 0, & {\rm{otherwise}}\end{array},$$

where ${{\mathscr{N}}}_{k}(i)$ represents the set including the $k$ nodes most similar to $i$, that is, $j$ is a nearest neighbour of $i$ if ${w}_{ij}$ is among the $k$ largest values in ${\{{w}_{ij}\}}_{\mathop{j=1,\ldots ,N}\limits_{j\ne i}}$.

The operation ${f}_{k}$ defines the adjacency matrix ${A}_{k}$ of the resulting unweighted graph: its $(i,j)$ element is ${A}_{k}(i,j)={f}_{k}({w}_{ij})$, with ${A}_{k}(i,i)=0$ as we do not allow for self-loops. Notice that ${f}_{k}$ does not preserve the symmetry, namely, in general,

2.9 $${A}_{k}(i,j)\ne {A}_{k}(j,i).$$

**Figure 12:**
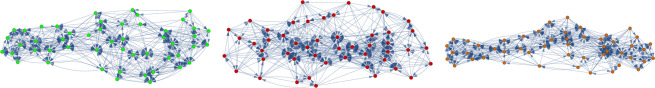
Examples of graphs for H (left), AF (center) and CD (right) patients. The graphs are realized by randomly extracting $N=50$ individuals in the H, AF and CD data-bases and by linking (with a directed edge) the first $k=10$ nearest patients for each node in the network according to the DTW distance.

Before proceeding, we stress that since we are now dealing with raw data, due to computational constraints, hereafter we focus solely on graphs made of $O\mathrm{(100)}$ nodes, examples of which are reported in Fig. 12.

#### Degree distributions

Due to the fact that the graphs ${{\mathscr{G}}}_{{\rm{H}},{\rm{AF}},{\rm{CD}}}$ are directed (see 2.9), we need to distinguish between the out-degree ${z}_{in}$ and the in-degree ${z}_{out}$ as

210 $${z}_{out}(i)=\mathop{\sum }\limits_{j=1}^{M}{A}_{k}(i,j),$$

211 $${z}_{in}(i)=\mathop{\sum }\limits_{j=1}^{M}{A}_{k}(j,i).$$

**Figure 13:**
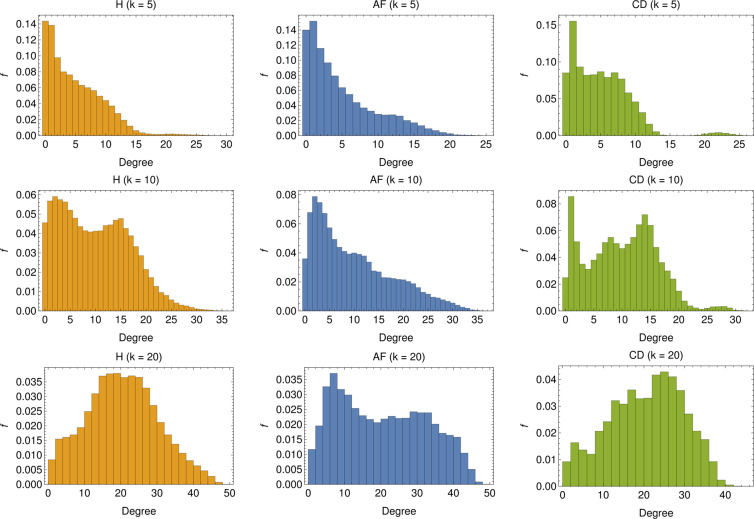
In-degree distribution for several choices of $k$: The distributions are realized by merging the degree distributions of 1000 different realizations of ${\mathscr{O}}\mathrm{(100)}$-node graphs. For low values of $k$ (i.e. $k=5$) we see that, for both the H and AF cases, the majority of nodes display rather low in-degree, and the in-degree distribution exhibits a long tail. Conversely, for the CD case, the in-degree is rather homogeneous up to ${d}_{in}\sim 10$, beyond which the tendency of nodes to acquire more links is softened (apart for a tiny fraction of nodes with ${d}_{in}\sim 20\div25$). By increasing $k$, nodes with low degree get fewer but still the most predominant for both H and AF patients but new peaks appear in the distribution (see $k=10$) highlighting a change of the topological structure of the network (see also the CD case). This structural change is clear for higher values of $k$ (i.e. $k=20$), especially for H and CD, for which low-linked nodes are fewer, the majority of nodes presenting an in-degree comparable with $k$ (${d}_{in}\sim 20\div25$), ultimately suggesting that the networks are becoming regular.

In other terms, the out-degree for the node $i$ is the number of nodes stemming from node $i$, while the in-degree is the number of links pointing to node $i$. The former is, by construction, equal to $k$, while the latter can vary (although its average is still equal to $k$): the in-degree for node $i$ is large if the related RR series is particularly similar to a large number of other series. The distribution for the in-degree is shown in Fig. 13, for several choices of $k$. In general, a broad distribution can be related to networks where the similarity relation between nodes, as established by Eq. 2.8, is far from symmetric. This seems to be the case especially in the networks ${{\mathscr{G}}}_{{\rm{AF}}}$ concerning AF patients.

**Figure 14:**
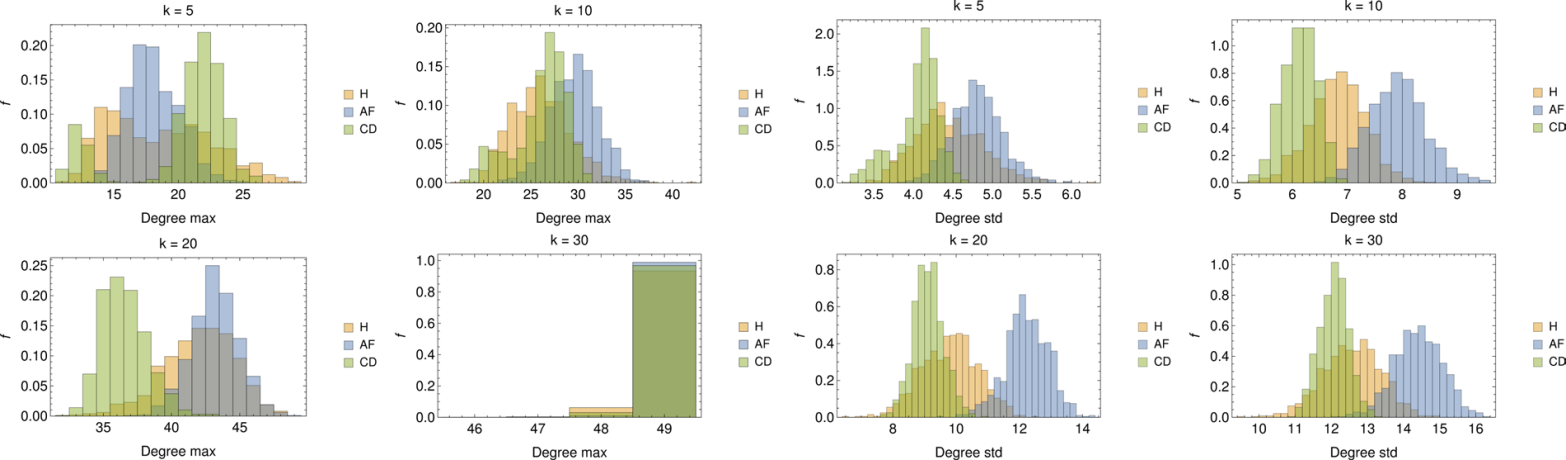
Left panel: Distributions of the maximal degree for values of $k\in (5,10,20,30)$. Apart for the largest value of $k$ (where the graph tends to a fully connected), in all the other cases, the average maximal degree seems able to split AF from CD patients aiming for proper classification. Right panel: Distributions of the degree standard deviations for values of $k\in (5,10,20,30)$; notice that, since for a certain choice of $k$, the average degree evaluated on different realizations of ${{\mathscr{G}}}_{{\rm{H}},{\rm{AF}},{\rm{CD}}}$ is constant and equal to $k$, the standard deviation correponds, a constant $k$ apart, to the variation coefficient.

Besides the degree distributions, we also study the maximum degree for each sample (see Fig. 14, left panel) and the degree standard deviations (see Fig. 14, right panel). As for the maximum in-degree, we see that for low values of $k$, its distribution for H patients is broad, for AF patients there are two distinct peaks (for ${d}_{max}\lesssim 15$ and ${d}_{max}\sim 20\div25$) tending to merge when increasing $k$ while, for CD patients, the distribution always presents the same shape, but with a different mean value; for $k\sim N/2$, in all of the three cases the most linked nodes are connected to all of the others in the graph. Finally, as for the degree standard deviations, recalling that for a given choice of $k$ the average in-degree is constant and equal to $k$, we can derive that, as expected, the broadness of the degree distribution decreases with $k$ and that ${{\mathscr{G}}}_{{\rm{AF}}}$ (resp. ${{\mathscr{G}}}_{{\rm{CD}}}$) displays the largest (resp. lowest) broadness. This further suggests that AF patients are relatively heterogeneous.

#### Clustering, reciprocities and cliques

The global clustering coefficient (GC) is a standard quantity in graph theory and it measures the fraction of closed triplets over the total number of triplets (both open and closed), that is, the likelihood that two neighbors of a node are neighbors themselves. For directed graphs, as those under study, it is convenient to define the CG as the fraction of closed path of length three over the total number of paths of the same length, where paths can only be taken in the allowed directions, that is,

2.12 $${\rm{GC}}=\frac{\#{\rm{allowed}}\,{\rm{closed}}\,{\rm{path}}\,{\rm{of}}\,{\rm{length}}\,{\rm{three}}}{\#{\rm{allowed}}\,{\rm{path}}\,{\rm{of}}\,{\rm{length}}\,{\rm{three}}}.$$

**Figure 15:**
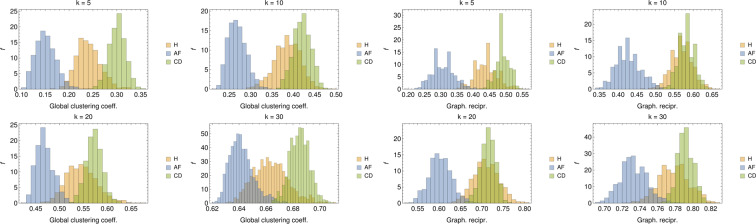
Left panel: Distributions of the GC coefficient for values of $k\in (5,10,20,30)$. For all values of $k$, also the global clustering coefficient seems able to discriminate among the various classes. In particular, systematically, AF patients share lower clustering while CD patients share higher values of clustering. Right panel: Distributions of the GR for values of $k\in (5,10,20,30)$. While the larger $k$ the more pronounced the overlap between H and CD patients, yet classification of AF patients seems robust.

The results for this quantifier are reported in Fig. 15 (left panel), where we show the histograms for the CG measured over 1000 different realizations of $O\mathrm{(100)}$-node graphs per class. As one can see, the distribution of global clustering coefficient is clearly different for the three populations (especially for low and high values of $k$). In particular, the GC measured in ${{\mathscr{G}}}_{{\rm{H}}}$ and ${{\mathscr{G}}}_{{\rm{AF}}}$ is on the average smaller than that obtained for ${{\mathscr{G}}}_{{\rm{CD}}}$. This suggests that, in the former cases, it is more likely for nodes to link with individuals across the whole network rather than in a restricted neighborhood, implying that the populations is heterogeneous (in agreement with the previous analysis on the degree distribution).

We now move to graph reciprocity (GR), which computes the reciprocal linkage of nodes in directed graphs: it is defined as the fraction of mutual (i.e. bidirectional, $i\to j$ and $j\to i$) links over the total number of edges in the graph, i.e.

2.13 $${\rm{GR}}=\frac{\#{\rm{reciprocal}}\,{\rm{links}}}{\#{\rm{links}}}.$$

Results for its distribution over the analyzed data-sets are reported in Fig. 15 (right panel) where it emerges that, for all values of $k$, AF patients have always a low degree of reciprocity suggesting that, in the corresponding adjacency matrix, ${A}_{k}(i,j)\ne {A}_{k}(j,i)$ for a large fraction of couples $(i,j)$ (such that nodes tend to link with other nodes rather then forming reciprocal bridges). At contrary, H and CD patients tend to link reciprocally in a similar way.

**Figure 16:**
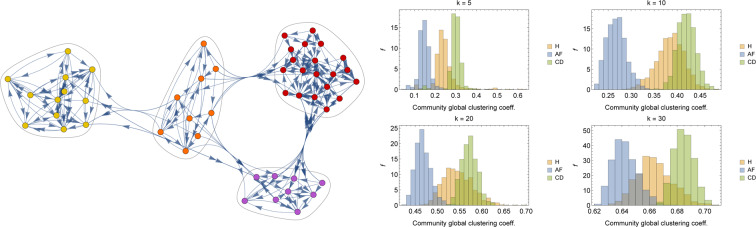
Left panel: A realization of ${{\mathscr{G}}}_{{\rm{H}}}$, where different communities are highlighted. Right panel: Distributions of the GC coefficient measured in the various community detected for values of $k\in (5,10,20,30)$. Also from this perspective, despite an increasing overlap between H and CD patients as $k$ grows, discrimination for the AF patients seems possible and robust.

Another interesting approach to determine emergent properties of a network is by community detection. More precisely, in this kind of analysis one aims to figure out the existence of groups of nodes, also called communities or clusters, displaying many edges joining nodes in the same group and comparatively few edges joining nodes of different groups. Detecting communities in large networks can be a hard problem and many algorithms have been proposed in the past years (see e.g.^53^); here, communities are detected with clique percolation methods^54,55^. An example of community detection in ${{\mathscr{G}}}_{{\rm{H}}}$ is reported in Fig. 16 (left panel). Further, the mean global clustering coefficient is measured in the various communities detected in 1000 different realization of $O\mathrm{(100)}$-node ${{\mathscr{G}}}_{{\rm{H}},{\rm{AF}},{\rm{CD}}}$ and the related distributions are reported in Fig. 16 (right panel). Also from this perspective, we see that AF patients present a generally lower value of mean community clustering coefficient, suggesting that neighbors in a given community do not tend to cluster among themselves.

## Discussion

Goal of the present study is the development of neural network models and machine learning algorithms that, given as input RR series, are able to discriminate between healthy versus cardiopath (atrial fibrillation or congestive heart failure) individuals. In particular, in the first part of the work, this task is framed into a multi-label classification problem tackled by a feed-forward neural network, with four possible outcomes: healthy (H), atrial fibrillation (AF), congestive heart failure (i.e., congestive decompensation CD), and other – not specified – pathologies (O). The classification is achieved on the basis of cardiological analysis: for each patient an Holter recording over a suitable time span of 24 h is available, from which standard clinical markers have been evaluated, resulting in a coarse-grained data-base containing the status of all the patients in terms of the values of these markers. Furthermore, in such a database, each patient is also provided with a label specifying the pathology it is suffering from (if any), allowing for supervised training of the network: the machinery developed turned out to successfully classify patients up to an accuracy ~$80\div85 \%$. It should be stressed that accuracy and, in general the network performance, could be further improved with a larger data-set. In the present case, the lack of a sufficiently large data-sets could be overcome with larger and larger on-line repositories where real data from routinely screened patients can be stored. This kind of policy has already been applied in several diagnostic fields (see e.g.^56^).

In the second part of the work, we investigated another possible route for disease classification that is based on network theory. More specifically, starting from the raw RR series and keeping the analysis independently split in the various classes of healthy patients, atrial fibrillation patients and congestive heart failure patients, we built class-related graphs by a standard similarity measure (the dynamical time warping) and then we inspected the emerging properties of these networks by studying standard topological features in network theory (e.g. degree distribution, global and local clustering coefficients, reciprocity and clique proliferation). Remarkably, even this route turned out to be successful in cardiac pathology classification, hence providing a complementary route to this purpose.

Overall the analysis carried on in this work evidenced that machine learning routes in cardiac pathology classification via HRV time-series analysis are possible and this may provide important benefits in terms of social costs: this should further prompt the establishment of shared repositories. Likewise, extensions of this approach to other pathologies could be feasible, as long as suitable experimental datasets are available.

## Apendix

### A Additional Information: a few details about the set of clinical markers

In this appendix we collect details about the markers considered in this work. First, in Tables 3 and 4, we report the full list of markers pertaining to the time domain and to the frequency domain, respectively; in Table 5, we report the full list of non-linear markers.

**Table 3:** List of linear markers (time domain), along with a synthetic description.

#	Marker	Synthetic description
1	Mean RR	Mean value of RR intervals
2	SDNN	Standard deviation of RR intervals
3	Mean HR	Mean value of BPM
4	STDHR	Standard deviation of instantaneous BPM
5	RMSSD	Square root of the mean squared differences between successive RR intervals
6	NN50	Number of successive RR interval pairs that differ more than 50 ms
7	pNN50	NN50 divided by the total number of RR intervals
8	HRV TIN	The integral of the RR interval histogram divided by the height of the histogram
9	TINN	Baseline width of the RR interval histogram
10	Mean RR5	Mean value of the standard deviations of the RR intervals in temporal windows of 5 minutes (RR5)
11	SDANN	Sample standard deviation of RR5 intervals in the total sampling time of 24 hours

**Table 4:** List of linear markers (frequency domain), along with a synthetic description.

#	Marker	Synthetic description
12	VLF peak (FFT)	Frequency peak in the VLF band (FFT-based methods)
13	Absolute Power VLF (FFT)	Absolute power of the VLF band (FFT-based methods)
14	Relative Power VLF (FFT)	Relative power of the VLF band (FFT-based methods)
15	LF peak (FFT)	Frequency peak in the LF band (FFT-based methods)
16	Absolute Power LF (FFT)	Absolute power of the LF band (FFT-based methods)
17	Relative Power LF (FFT)	Relative power of the LF band (FFT-based methods)
18	Normalized Power LF (FFT)	Normalized power of the LF band (FFT-based methods)
19	HF peak (FFT)	Frequency peak in the HF band (FFT-based methods)
20	Absolute Power HF (FFT)	Absolute power of the HF band (FFT-based methods)
21	Relative Power HF (FFT)	Relative power of the HF band (FFT-based methods)
22	Normalized Power HF (FFT)	Normalized power of the HF band (FFT-based methods)
23	Total Power (FFT)	Total power (FFT-based methods)
24	LH/HF (FFT)	LF/HF peaks ratio (FFT-based methods)
25	VLF peak (AR)	Frequency peak in the VLF band (autoregressive methods)
26	Absolute Power VLF (AR)	Absolute power of the VLF band (autoregressive methods)
27	Relative Power VLF (AR)	Relative power of the VLF band (autoregressive methods)
28	LF peak (AR)	Frequency peak in the LF band (autoregressive methods)
29	Absolute Power LF (AR)	Absolute power of the LF band (autoregressive methods)
30	Relative Power LF (AR)	Relative power of the LF band (autoregressive methods)
31	Normalized Power LF (AR)	Normalized power of the LF band (autoregressive methods)
32	HF peak (AR)	Frequency peak in the HF band (autoregressive methods)
33	Absolute Power HF (AR)	Absolute power of the HF band (autoregressive methods)
34	Relative Power HF (AR)	Relative power of the HF band (autoregressive methods)
35	Normalized Power HF (AR)	Normalized power of the HF band (autoregressive methods)
36	Total Power (AR)	Total power (autoregressive methods)
37	LF/HF (AR)	LF/HF peaks ratio (autoregressive methods)

**Table 5:** List of non-linear markers, along with a synthetic description provided.

#	Marker	Synthetic description
38	SD1	Standard deviation of the Poincaré plot in the orthogonal direction to the identity line
39	SD2	Standard deviation of the Poincaré plot along the identity line
40	RPL mean	Mean line length of diagonal lines in Recurrence Plot
41	RPL max	Maximum line length of diagonal lines in Recurrence Plot
42	REC	Recurrence rate (percentage of recurrence points in Recurrence Plot)
43	DET	Determinism (percentage of recurrence points which form diagonal lines in Recurrence Plot)
44	ShanEn	Shannon Entropy of diagonal line lenghts’ probability distribution
45	ApEn	Approximate entropy
46	SampEn	Sample entropy
47	DFA ${\alpha }_{1}$	Short-term fluctuations of Detrended Fluctuation Analysis
48	DFA ${\alpha }_{2}$	Long-term fluctuations of Detrended Fluctuation Analysis
49	D2	Correlation dimension

**Figure 17:**
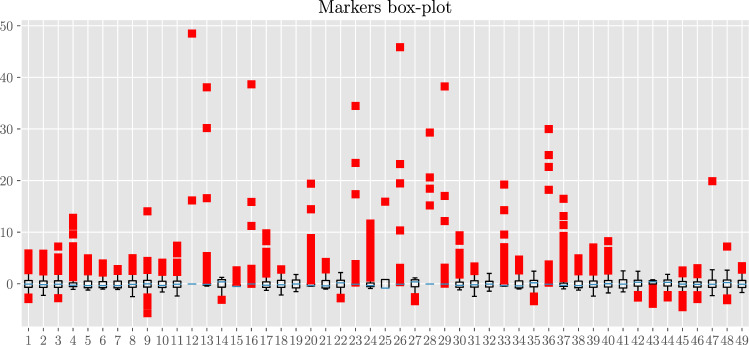
Box-plot diagram for the 49 standardized markers. Each line corresponds to a different marker as highlighted by the index on the left (see also Table 1). The blue vertical line within each box denotes the related median and each box spans from the lower to the upper related quartiles. Outer bars (whiskers) range from the lowest to the highest non-outlier data points. Bullets correspond to outlier data points.

In Fig. 17 we present the box-plot for the standardized markers. As expected (given that the statistics underlying HRV is heavy-tailed), there is a large presence of outlier points highlighting the broadness in the marker distributions.

Ethical Statement 1: Informed consent was obtained from all subjects and related data have been treated in a completely anonymous form (in the full respect of the *Declaration of Helsinki* (1964) with the understanding and the consent of the human subjects involved in the study).

Ethical Statement 2: APH and POLISA asked for explicit approval of the study by the responsible Ethical Committee: this approval was released to APH and POLISA on June 09 2016 by the Ethical Committee of Regione Marche (APH Hospital belongs to that region) and can be supplied upon request.

Etichal Statement 3: all the methods were carried out in strick accordance with all the relative guidelines and regulations in Italy.
